# [*N*,*N*′-Bis(4-chlorobenzylidene)-2,2-dimethylpropane-1,3-diamine-κ^2^ *N,N*′]iodidocopper(I)

**DOI:** 10.1107/S160053680900107X

**Published:** 2009-01-17

**Authors:** Reza Kia, Hoong-Kun Fun, Hadi Kargar

**Affiliations:** a X-ray Crystallography Unit, School of Physics, Universiti Sains Malaysia, 11800 USM, Penang, Malaysia; b Department of Chemistry, School of Science, Payame Noor University (PNU), Ardakan, Yazd, Iran

## Abstract

The mol­ecule of the title compound, [CuI(C_19_H_20_Cl_2_N_2_)], lies across a crystallographic mirror plane. The coordination around the copper centre is distorted trigonal planar, with a bite angle of 94.40 (7)°. A six-membered chelate ring is formed by the coordination of iminic N atoms of the bidentate ligand to the Cu^I^ atom, adopting a chair conformation. This conformation is required if the local symmetry of the metal coordination site is in accordance with a mirror plane that passes through the metal atom normal to the line connecting the N atoms. The dihedral angle between the benzene rings is 78.66 (5)°. The crystal structure is stabilized by weak inter­molecular C—H⋯π inter­actions, which link the mol­ecules into chains along the *b* axis.

## Related literature

For puckering parameters, see: Cremer & Pople (1975 ▶). For related literature and the catalytic applications, see, for example: Killian *et al.* (1996 ▶); Jung *et al.* (1996 ▶); Small *et al.* (1998 ▶). For hydrogen-bond motifs, see: Bernstein *et al.* (1995 ▶). 
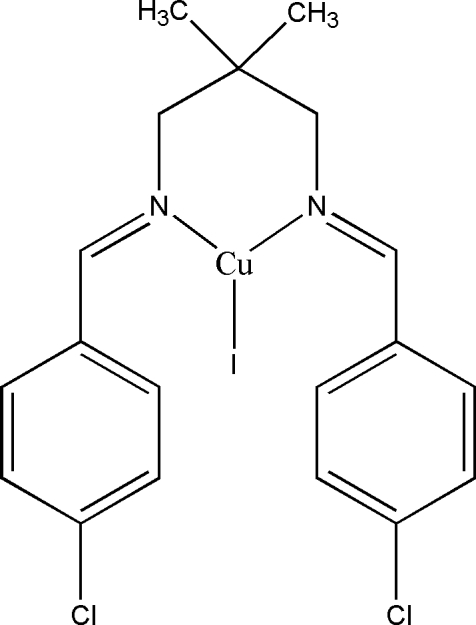


## Experimental

### 

#### Crystal data

[CuI(C_19_H_20_Cl_2_N_2_)]
*M* *_r_* = 537.71
Monoclinic, 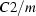

*a* = 16.2770 (1) Å
*b* = 12.2983 (1) Å
*c* = 10.6255 (1) Å
β = 92.249 (1)°
*V* = 2125.37 (3) Å^3^
*Z* = 4
Mo *K*α radiation
μ = 2.74 mm^−1^
*T* = 296 (2) K
0.44 × 0.31 × 0.28 mm

#### Data collection

Bruker APEXII CCD area-detector diffractometer
Absorption correction: multi-scan (**SADABS**; Bruker, 2005 ▶) *T*_min_ = 0.334, *T*_max_ = 0.463
39802 measured reflections
4869 independent reflections
4157 reflections with *I* > 2σ(*I*)
*R*_int_ = 0.026

#### Refinement

*R*[*F* ^2^ > 2σ(*F* ^2^)] = 0.024
*wR*(*F* ^2^) = 0.067
*S* = 1.02
4869 reflections
122 parameters
H-atom parameters constrained
Δρ_max_ = 0.74 e Å^−3^
Δρ_min_ = −0.55 e Å^−3^

### 

Data collection: *APEX2* (Bruker, 2005 ▶); cell refinement: *SAINT* (Bruker, 2005 ▶); data reduction: *SAINT*; program(s) used to solve structure: *SHELXTL* (Sheldrick, 2008 ▶); program(s) used to refine structure: *SHELXTL*; molecular graphics: *SHELXTL*; software used to prepare material for publication: *SHELXTL* and *PLATON* (Spek, 2003 ▶).

## Supplementary Material

Crystal structure: contains datablocks global, I. DOI: 10.1107/S160053680900107X/hk2609sup1.cif

Structure factors: contains datablocks I. DOI: 10.1107/S160053680900107X/hk2609Isup2.hkl

Additional supplementary materials:  crystallographic information; 3D view; checkCIF report

## Figures and Tables

**Table 1:** Hydrogen-bond geometry (Å, °)

*D*—H⋯*A*	*D*—H	H⋯*A*	*D*⋯*A*	*D*—H⋯*A*
C8—H8*A*⋯*Cg*1^i^	0.97	2.92	3.7220 (19)	141

Symmetry code: (i) 
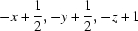
. *Cg*1 is the centroid of the C1–C6 benzene ring.

**Fig. 1.**
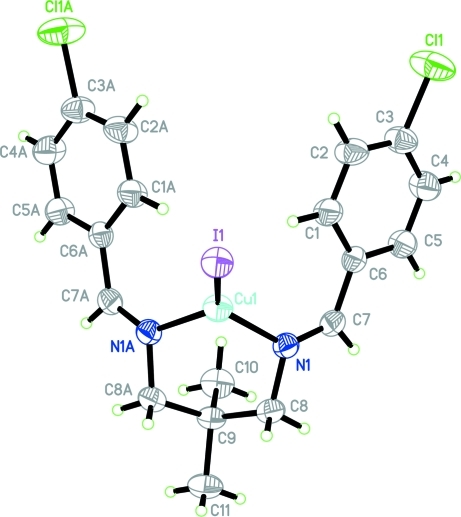
The molecular structure of (I), showing 40% probability displacement ellipsoids and the atomic numbering. Symmetry code for A atoms; X, -Y, Z.

**Fig. 2.**
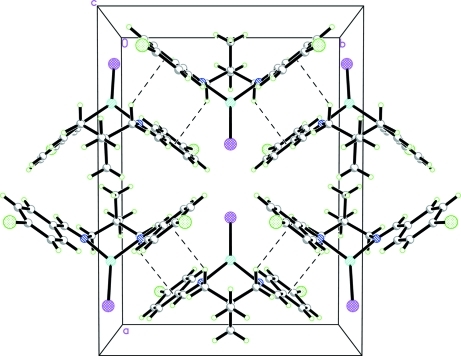
The crystal packing of (I), viewed down the c-axis, showing C—H···π interactions linking the molecules into chains along the b-axis.

## supplementary crystallographic information

### Comment

In recent years, an increasing amount of research has been focused on the design and preparation of mono- or di-nuclear mixed ligand transition metal complexes containing neutral, chelating nitrogen ligands. Early and late transition metal complexes of this type have extensively been used as catalysts for a wide categories of reactions, including olefin polymerization (Killian *et al.,* 1996) and oxygen activation (Jung *et al.,* 1996). In this context, diverse chelating Schiff base type ligands, amines and pyridine derivatives (Small *et al.,* 1998) have successfully been applied in the preparation of these homogeneous catalysts. Here we report the crystal structure of an aldimine Schiff base ligand with copper(I) iodide. To the best of our knowledge, the title compound is the first tricoordinate complex of an aldimine bis-Schiff base ligand with copper(I) iodide adopting triginal planar geometry.

The title compound, I, Fig. 1, lies across a crystallographic mirror plane. Atoms I1, Cu1, C9, C10 and C11 lies on this mirror plane. The asymmetric unit of (I) is composed of one-half of the molecule. The coordination geometry around copper has a distorted trigonal planar geometry. The deviation of the Cu atom from the N1/N1A/I1 plane is -0.1213 (8) Å. A six-membered chelate ring is formed in this case by the coordination of iminic nitrogen atoms of the bidentate ligand which adopts the chair conformation with the ring puckering paremeters (Cremer & Pople 1975) of Q = 0.7001 (14) Å, Θ = 7.72 (11)°, Φ = 0.0 (9)°. This conformation is required if the local symmetry of the metal coordination site is in accordance with a mirror plane that passed through the metal atom normal to the line connecting the nitrogen atoms. The dihedral angle between the phenyl rings is 78.66 (5)°. The crystal structure is stabilized by weak intermolecular C—H···π interactions (*Cg*1 is the centroid of the C1–C6 benzene ring) which link the molecules into chains along the *b*-axis (Fig. 2 and Table 1).

### Experimental

*N,N'*-Bis(4-chlorobenzylidene)-2,2-dimethylpropane (694 mg, 2 mmol) was added dropwise to a suspension of CuI (380 mg, 2.0 mmol) in 50 ml of THF. After 15 minutes a clear yellowish solution was obtained. The volume of the reaction mixture was reduced until the formation of a yellow precipitate occurred. Single crystals suitable for X-ray diffraction were grown from the acetonitrile solution.

### Refinement

All H atoms were positioned geometrically with C—H = 0.93 Å (aromatic), 0.96 Å (methyl), and 0.97 Å (methylene) and refined in the riding model approximation with *U*_iso_(H) = 1.2 or 1.5 *U*_eq_(C). The highest peak (0.74 e. Å^-3^) is located 0.60 Å from I1 and the deepest hole (-0.55 e. Å^-3^ is located 0.59 Å from I1.

### Crystal data

**Table:**

[CuI(C_19_H_20_Cl_2_N_2_)]	*F*(000) = 1056
*M**_r_* = 537.71	*D*_x_ = 1.674 Mg m^−^^3^
Monoclinic, *C*2/*m*	Mo *K*α radiation, λ = 0.71073 Å
Hall symbol: -C 2y	Cell parameters from 9801 reflections
*a* = 16.2770 (1) Å	θ = 2.5–35.7°
*b* = 12.2983 (1) Å	µ = 2.74 mm^−^^1^
*c* = 10.6255 (1) Å	*T* = 296 K
β = 92.249 (1)°	Block, yellow
*V* = 2125.37 (3) Å^3^	0.44 × 0.31 × 0.28 mm
*Z* = 4	

### Data collection

**Table:**

Bruker SMART APEXII CCD area-detector diffractometer	4869 independent reflections
Radiation source: fine-focus sealed tube	4157 reflections with *I* > 2σ(*I*)
graphite	*R*_int_ = 0.026
φ and ω scans	θ_max_ = 35.0°, θ_min_ = 1.9°
Absorption correction: multi-scan (*SADABS*; Bruker, 2005)	*h* = −26→26
*T*_min_ = 0.334, *T*_max_ = 0.463	*k* = −19→19
39802 measured reflections	*l* = −17→17

### Refinement

**Table:**

Refinement on *F*^2^	Secondary atom site location: difference Fourier map
Least-squares matrix: full	Hydrogen site location: inferred from neighbouring sites
*R*[*F*^2^ > 2σ(*F*^2^)] = 0.024	H-atom parameters constrained
*wR*(*F*^2^) = 0.067	*w* = 1/[σ^2^(*F*_o_^2^) + (0.031*P*)^2^ + 1.3061*P*] where *P* = (*F*_o_^2^ + 2*F*_c_^2^)/3
*S* = 1.02	(Δ/σ)_max_ < 0.001
4869 reflections	Δρ_max_ = 0.74 e Å^−^^3^
122 parameters	Δρ_min_ = −0.55 e Å^−^^3^
0 restraints	Extinction correction: *SHELXL*, Fc^*^=kFc[1+0.001xFc^2^λ^3^/sin(2θ)]^-1/4^
Primary atom site location: structure-invariant direct methods	Extinction coefficient: 0.00458 (18)

### Special details

**Table:**

Experimental. The low-temperature data were collected with the Oxford Cyrosystem Cobra low-temperature attachment
Geometry. All esds (except the esd in the dihedral angle between two l.s. planes) are estimated using the full covariance matrix. The cell esds are taken into account individually in the estimation of esds in distances, angles and torsion angles; correlations between esds in cell parameters are only used when they are defined by crystal symmetry. An approximate (isotropic) treatment of cell esds is used for estimating esds involving l.s. planes.
Refinement. Refinement of F^2^ against ALL reflections. The weighted R-factor wR and goodness of fit S are based on F^2^, conventional R-factors R are based on F, with F set to zero for negative F^2^. The threshold expression of F^2^ > 2sigma(F^2^) is used only for calculating R-factors(gt) etc. and is not relevant to the choice of reflections for refinement. R-factors based on F^2^ are statistically about twice as large as those based on F, and R- factors based on ALL data will be even larger.

### Fractional atomic coordinates and isotropic or equivalent isotropic displacement parameters (Å^2^)

**Table:**

	*x*	*y*	*z*	*U*_iso_*/*U*_eq_	
I1	0.116357 (8)	0.0000	0.351624 (13)	0.04474 (5)	
Cu1	0.250035 (16)	0.0000	0.46811 (2)	0.04325 (7)	
Cl1	0.38586 (4)	0.32893 (7)	−0.03450 (5)	0.0900 (2)	
N1	0.31811 (7)	0.11994 (10)	0.54747 (11)	0.0410 (2)	
C1	0.32263 (11)	0.17020 (14)	0.27282 (15)	0.0515 (4)	
H1A	0.2889	0.1119	0.2920	0.062*	
C2	0.32963 (11)	0.20089 (16)	0.14902 (16)	0.0567 (4)	
H2A	0.3009	0.1637	0.0851	0.068*	
C3	0.37946 (11)	0.28682 (17)	0.12088 (16)	0.0545 (4)	
C4	0.42404 (12)	0.34113 (16)	0.21435 (18)	0.0603 (4)	
H4A	0.4593	0.3974	0.1940	0.072*	
C5	0.41577 (11)	0.31124 (14)	0.33842 (16)	0.0519 (3)	
H5A	0.4444	0.3493	0.4017	0.062*	
C6	0.36540 (9)	0.22526 (11)	0.37029 (13)	0.0411 (3)	
C7	0.35971 (9)	0.19805 (12)	0.50364 (14)	0.0440 (3)	
H7A	0.3889	0.2415	0.5614	0.053*	
C8	0.32286 (11)	0.10423 (13)	0.68508 (14)	0.0486 (3)	
H8A	0.2675	0.1019	0.7156	0.058*	
H8B	0.3505	0.1664	0.7237	0.058*	
C9	0.36849 (14)	0.0000	0.72732 (19)	0.0456 (4)	
C10	0.45648 (16)	0.0000	0.6822 (3)	0.0618 (6)	
H10A	0.4554	0.0000	0.5918	0.093*	
H10B	0.4847	−0.0637	0.7132	0.093*	
C11	0.3698 (2)	0.0000	0.8725 (2)	0.0717 (8)	
H11A	0.3144	0.0000	0.9003	0.108*	
H11B	0.3978	0.0637	0.9038	0.108*	

### Atomic displacement parameters (Å^2^)

**Table:**

	*U*^11^	*U*^22^	*U*^33^	*U*^12^	*U*^13^	*U*^23^
I1	0.03731 (7)	0.05062 (8)	0.04559 (8)	0.000	−0.00706 (5)	0.000
Cu1	0.03802 (12)	0.05125 (14)	0.04003 (12)	0.000	−0.00415 (9)	0.000
Cl1	0.0925 (4)	0.1280 (6)	0.0491 (2)	−0.0450 (4)	−0.0017 (2)	0.0181 (3)
N1	0.0443 (6)	0.0406 (5)	0.0381 (5)	0.0015 (4)	−0.0004 (4)	−0.0020 (4)
C1	0.0583 (9)	0.0507 (8)	0.0454 (7)	−0.0190 (7)	0.0027 (6)	−0.0049 (6)
C2	0.0583 (9)	0.0682 (10)	0.0433 (7)	−0.0221 (8)	0.0003 (6)	−0.0066 (7)
C3	0.0507 (8)	0.0683 (10)	0.0444 (7)	−0.0135 (7)	0.0022 (6)	0.0042 (7)
C4	0.0625 (10)	0.0636 (10)	0.0548 (9)	−0.0268 (8)	0.0007 (7)	0.0043 (8)
C5	0.0539 (8)	0.0520 (8)	0.0492 (8)	−0.0170 (7)	−0.0044 (6)	−0.0023 (6)
C6	0.0416 (6)	0.0381 (6)	0.0435 (6)	−0.0026 (5)	−0.0005 (5)	−0.0035 (5)
C7	0.0496 (7)	0.0396 (6)	0.0425 (6)	−0.0034 (5)	−0.0019 (5)	−0.0060 (5)
C8	0.0578 (8)	0.0509 (8)	0.0372 (6)	0.0027 (6)	0.0037 (6)	−0.0045 (6)
C9	0.0502 (11)	0.0543 (11)	0.0320 (8)	0.000	0.0000 (7)	0.000
C10	0.0463 (12)	0.0775 (17)	0.0611 (14)	0.000	−0.0050 (10)	0.000
C11	0.102 (2)	0.0794 (19)	0.0336 (10)	0.000	−0.0029 (12)	0.000

### Geometric parameters (Å, °)

**Table:**

I1—Cu1	2.4607 (3)	C5—C6	1.388 (2)
Cu1—N1	2.0104 (12)	C5—H5A	0.9300
Cu1—N1^i^	2.0104 (12)	C6—C7	1.462 (2)
Cl1—C3	1.7373 (17)	C7—H7A	0.9300
N1—C7	1.2739 (19)	C8—C9	1.540 (2)
N1—C8	1.4739 (18)	C8—H8A	0.9700
C1—C2	1.378 (2)	C8—H8B	0.9700
C1—C6	1.3998 (19)	C9—C10	1.528 (3)
C1—H1A	0.9300	C9—C8^i^	1.540 (2)
C2—C3	1.372 (2)	C9—C11	1.542 (3)
C2—H2A	0.9300	C10—H10A	0.9600
C3—C4	1.379 (2)	C10—H10B	0.9601
C4—C5	1.380 (2)	C11—H11A	0.9600
C4—H4A	0.9300	C11—H11B	0.9600
			
N1—Cu1—N1^i^	94.40 (7)	C1—C6—C7	123.89 (13)
N1—Cu1—I1	132.30 (3)	N1—C7—C6	125.56 (13)
N1^i^—Cu1—I1	132.30 (3)	N1—C7—H7A	117.2
C7—N1—C8	116.98 (13)	C6—C7—H7A	117.2
C7—N1—Cu1	133.79 (10)	N1—C8—C9	113.84 (13)
C8—N1—Cu1	109.06 (10)	N1—C8—H8A	108.8
C2—C1—C6	121.10 (14)	C9—C8—H8A	108.8
C2—C1—H1A	119.4	N1—C8—H8B	108.8
C6—C1—H1A	119.4	C9—C8—H8B	108.8
C3—C2—C1	119.40 (15)	H8A—C8—H8B	107.7
C3—C2—H2A	120.3	C10—C9—C8^i^	110.85 (12)
C1—C2—H2A	120.3	C10—C9—C8	110.85 (12)
C2—C3—C4	120.98 (16)	C8^i^—C9—C8	112.74 (19)
C2—C3—Cl1	119.64 (13)	C10—C9—C11	109.8 (2)
C4—C3—Cl1	119.38 (13)	C8^i^—C9—C11	106.21 (13)
C3—C4—C5	119.38 (15)	C8—C9—C11	106.21 (13)
C3—C4—H4A	120.3	C9—C10—H10A	109.5
C5—C4—H4A	120.3	C9—C10—H10B	109.5
C4—C5—C6	121.10 (14)	H10A—C10—H10B	109.5
C4—C5—H5A	119.5	C9—C11—H11A	109.4
C6—C5—H5A	119.5	C9—C11—H11B	109.5
C5—C6—C1	118.00 (14)	H11A—C11—H11B	109.5
C5—C6—C7	118.11 (13)		

Symmetry codes: (i) *x*, −*y*, *z*.

### Hydrogen-bond geometry (Å, °)

**Table:**

*D*—H···*A*	*D*—H	H···*A*	*D*···*A*	*D*—H···*A*
C8—H8A···Cg1^ii^	0.97	2.92	3.7220 (19)	141

Symmetry codes: (ii) −*x*+1/2, −*y*+1/2, −*z*+1.

## References

[bb1] Bernstein, J., Davis, R. E., Shimoni, L. & Chamg, N.-L. (1995). *Angew. Chem. Int. Ed. Engl.* **34**, 1555–1573.

[bb2] Bruker (2005). *APEX2*, *SAINT* and *SADABS* Bruker AXS Inc., Madison, Wisconsin, USA.

[bb3] Cremer, D. & Pople, J. A. (1975). *J. Am. Chem. Soc.* **97**, 1354–1358.

[bb4] Jung, B., Karlin, K. D. & Zuberbühler, A. D. (1996). *J. Am. Chem. Soc.*, **118**, 3763–3768.

[bb5] Killian, C. M., Tempel, D. J., Johnson, L. K. & Brookhart, M. (1996). *J. Am. Chem. Soc.* **118**, 11664–11670.

[bb6] Sheldrick, G. M. (2008). *Acta Cryst.* A**64**, 112–122. 10.1107/S0108767307043930 18156677

[bb7] Small, B. L., Brookhart, M. & Bennett, A. M. A. (1998). *J. Am. Chem. Soc.*, **120**, 4049–4054.

[bb8] Spek, A. L. (2003). *J. Appl. Cryst.* **36**, 7–13.

